# Evaluation of Cocoa Beans Shell Powder as a Bioadsorbent of Congo Red Dye Aqueous Solutions

**DOI:** 10.3390/ma14112763

**Published:** 2021-05-23

**Authors:** Gabriela Rodríguez-Arellano, Juan Barajas-Fernández, Ricardo García-Alamilla, Laura Mercedes Lagunes-Gálvez, Antonio Hilario Lara-Rivera, Pedro García-Alamilla

**Affiliations:** 1 Academic División of Engineering and Architecture (DAIA), Juarez Autonomous University of Tabasco (UJAT), Carretera Cunduacán-Jalpa KM. 1, Col. La Esmeralda, Cunduacán C.P. 86690, Tabasco, Mexico; gaby_girl22@hotmail.com (G.R.-A.); juan.barajas@ujat.mx (J.B.-F.); 2 National Technological Institute of Mexico/I.T.CD. Madero, Petrochemical Research Center, Prol. Bahía de Aldahir y Av. De las Bahías, Parque de la Pequeña y Mediana Industria, Altamira 89600, Tamaulipas, Mexico; ricardo.ga@cdmadero.tecnm.mx; 3 Academic División of Agriculture Science (DACA), Juarez Autonomous University of Tabasco (UJAT), Carretera Villahermosa-Teapa km. 25, Ranchería La Huasteca 2da. Sección, Centro C.P. 86280, Tabasco, Mexico; laura.lagunes@ujat.mx; 4 Center of Agricultural Technological Baccalaureate no. 293, “Ing. Edmundo Taboada Ramírez”, Zapotlán El Grande, Jalisco, Carretera Ciudad Guzmán-El Grullo km. 105, Parque Industrial Zapotlán 2000, Ciudad Guzmán C.P. 49000, Jalisco, Mexico; alarin79@hotmail.com

**Keywords:** environmental impact, dye degradation, bioadsorbent, cocoa bean shell, Congo red adsorption, response surface methodology

## Abstract

The use of synthetic dyes in the textile, leather, and paper industries is a source of groundwater pollution around the world. There are different methods for the treatment of wastewater that has been contaminated with dyes, among which adsorption with agro-industrial wastes is gaining relevance. In the present study, the adsorption capacity of cocoa bean shell powder was evaluated when it was used as a bioadsorbent for Congo red dye in an aqueous medium. A 2^4^ central factorial design with central and axial points was proposed to determine the adsorption capacity. The factors that were studied were the adsorbent (0.06–0.15 g), Congo red (40–120 mg L^−1^), pH (3–11), and time (4–36 h). The bioadsorbent was characterized through scanning electron microscopy and Fourier-transform infrared spectroscopy. The effects of the factors on the adsorption capacity for Congo red using cocoa bean shell were nonlinear, and they were modeled with a second-order polynomial (*p* < 0.05) and with an R^2^ of 0.84. The bioadsorbent obtained a maximum adsorption of 89.96% in runs. The process of optimization by using the surface response allowed the maximization of the adsorption, and the validation showed that 95.79% adsorption of the dye was obtained.

## 1. Introduction

Anthropogenic activity resulting from population growth and industrial development has deteriorated the environment and, consequently, the quality of water [1,2,3]. The processing of raw materials into finished products requires large amounts of water, which are discharged into seas and rivers after processing, with varying degrees of contamination. In particular, the water contaminated by synthetic dyes comes from various industrial activities; among them, the food, leather, textile, paper, medicine, cosmetic, printing-ink, varnish, paint, oil, and soap industries stand out [1,2,3,4,5]. The textile and food industries alone dispose of between 10% and 20% of the contaminants that impart color to water during their production processes [6], thus constituting a challenge for water treatment [7]. According to Bansal et al. [1], more than 7 million tons of synthetic dyes and colorants are produced each year by various industries. In addition, 10,000 different types of colorants are accessible according to the color index, and more than 10,000 viable colorants are manufactured each year for industrial purposes. The classification of colorants divides them into anionic, cationic, and nonionic, each containing a large number of dyes. Dyes have a high molecular weight and are very difficult to degrade because there many obstacles that must be overcome, such as their complex structures and stability [3,8].

In the first place, contamination by synthetic dyes has a great visual impact, and secondly, the dyes are mostly organic molecules that are toxic for aquatic life [9,10,11]. Their main effect as pollutants is that they limit photosynthetic activity as a result of the decrease in light, causing the death of flora [12]. In addition, the oxidation of the dye consumes dissolved oxygen, so it directly affects the respiratory activity of aquatic organisms [4]. Due to their mostly aromatic molecular structures, synthetic dyes are recalcitrant to biodegradation [13]. Finally, the long-term contamination of water supplies aggravates the mutagenic, carcinogenic, and pathogenic potential in humans [4]. The population has become aware of the problem of water pollution, and different organizations have issued regulations; therefore, there is a growing demand for the development of wastewater technology [1].

Recently, several methods have been studied for the treatment of effluents contaminated with dyestuffs, such as filtration; precipitation; photochemical degradation; chemical coagulation; electrocoagulation; photocatalysis; ultrasonic, biological, or enzymatic treatment; and chemical oxidation [1,3,4]. However, even in efficient cases, these technologies have severe limitations for their implementation in addition to their high costs [14,15]. The above problems justify the search for new alternatives for the remediation of effluents for which the economic aspects and technical competitiveness can be highlighted.

Adsorption has been employed since ancient times as an alternative for water cleaning, mainly by employing carbon. The removal of synthetic dyes by employing agro-food wastes as bioadsorbents is an area of research and development, as these are effective and highly economical [2,16]. In recent years, much emphasis has been placed on the fact that industrial, forestry, and agro-industrial wastes have a low cost due to their abundant disposal, and they require little processing, which makes them attractive for such applications [3,17,18]. The biomass obtained from such wastes has been used to generate extracts that are used as adsorbents and to prepare nanomaterials for the same purpose [3].

The agricultural wastes that have been most studied as adsorbents are rice shells, banana shells, wheat shells, tamarind shells, sugarcane bagasse, papaya seeds, and pumpkin seeds [19,20]. Within this context, recent reports from the Secretariat of Agriculture, Livestock, Rural Development, Fisheries, and Food (SAGARPA) of Mexico indicated that 26,866 tons of cocoa, an emblematic crop of the state of Tabasco, were produced in 2016. The cocoa bean has been successfully commercialized for the development of derivative products of great commercial value—mainly chocolate—but after the roasting process, the cocoa bean shell is an agro-industrial waste product of very little commercial value. According to Arlorio et al. [21] and Lecumberri et al. [22], the cocoa bean shell contains, on average, 167 to 181 g kg^−1^ dm^−1^ of protein, 66 to 68 g kg^−1^ dm^−1^ of fat, 81 to 114 g kg^−1^ dm^−1^ of ash, and mainly 605 to 606 g kg^−1^ dm^−1^ of dietary fiber. The fiber is made up of lignin and cellulose, resulting in the appearance of hemicellulose and adhered polysaccharides at a higher level. Because of the high fiber content, the cocoa bean shell is presented as an alternative bioadsorbent. The uses of cocoa bean shells for extracting bioactive compounds in order to give added value have been studied, but the reports are at the research level. The cocoa bean shell produces valuable extracts that are rich in antioxidant flavanols (catechins and epicatechins), theobromine, caffeine, and cocoa butter [23,24].

The main contribution of this work is the evaluation of the adsorption of Congo red dye (CR) by using the shell adhered to the cocoa bean as a bioadsorbent. CR is a diazo dye with thermal, physicochemical, and optical stability, as well as high toxicity for living species; for this reason, it is used as a model molecule for studies where adsorbent agents are tested.

To achieve the proposed objective, the response surface methodology (RSM) was used; this is the most commonly applied technique for evaluating the effects of independent variables on a process. The RSM combines mathematics and statistics based on a polynomial equation established based on experimental data that are defined through a factorial design [2,9]. In the process of dye adsorption in an aqueous medium, the effects of the main variables affecting the process in question were studied, such as the dye concentration, the amount of adsorbent, the pH, and the contact time. A 2^4^ factorial design with central and axial points was used to determine the combinations in the adsorption treatments. The response surface methodology (RSM) was applied to generate a polynomial response, which allowed the maximization of the adsorption of Congo red through the optimization of the study factors and the experimental validation of the response; 95.79% adsorption of the dye was achieved.

## 2. Materials and Methods

### 2.1. Conditioning of the Bioadsorbent Material

The shell that covers the cocoa bean was used as a raw material in the preparation of the bioadsorbent powder for Congo red dye. The shell was obtained after the cocoa bean roasting process, an industrial stage of processing in which the shell is separated from the cotyledon and the germ. The shell was washed with abundant water and exposed directly to the sunlight until it was dried. The dried shell was ground in a multifunctional coffee mill (Krups GX4100, Mexico D.F., Mexico), and the powder was passed through a no. 20 mesh sieve (Montinox, Montiel, Pantitlan, Mexico). The recovered powder with homogeneous particle size was washed with distilled water and then with deionized water until the sample no longer released its natural color. Finally, the powder was vacuum-filtered and subjected to a drying process in an oven (Felisa FE-291, Guadalajara, Mexico) for 3 h. It was crushed again and sieved to recover the powder that passed through the no. 50 mesh sieve, thus obtaining a bioadsorbent with a particle size of less than 297 μm.

### 2.2. Adsorption Studies

The efficiency of the adsorbent (cocoa bean shell powder) for dye removal was evaluated through batch experimentation. Solutions of Congo red dye (Sigma Aldrich, Burlington, MA, USA) were prepared in distilled water with different concentrations (40 to 120 mg L^−1^). The solutions were modified in terms of their pH (3 to 11) by adding 0.1 N of sodium hydroxide (NaOH) and hydrochloric acid (HCl) as required. The adsorption experiments were performed in a 250 mL batch reactor, where 0.06 to 0.18 g of the adsorbent was added. The number of runs was established according to the experimental design described below. The suspension of the dye and bioadsorbent under study was placed in an orbital shaker (Thermo Fisher Scientific, Multipurpose rotator, 2346, Assembled China) at 120 rpm for different contact times (4 to 36 h). At the end of the assay, aliquots of the process were taken and subjected to centrifugation (Hermle Labortechnik GmbH type: Z326K, Wehingen, Germany) at 5000 rpm for 10 min at room temperature. An aliquot of the supernatant was taken, and the absorbance of the samples was read at 490 nm in a spectrophotometer (GENESYS 10S UV-Vis, Thermo Scientific, Assembled China); according to the calibration curve (2–10 mg L^−1^), the final concentration of the samples was obtained.

### 2.3. Assessment of the Adsorption Capacity

From the data obtained from the adsorption experiments, the percentage of dye removal (% A) was calculated using the following equation:

(1) $$\%\;A=\frac{C_{i}-C_{f}}{C_{i}}\times 100,$$

where *C_i_* is the initial concentration and *C_f_* is the final concentration in the solution.

### 2.4. Characterization of the Bioadsorbent

#### 2.4.1. Scanning Electron Microscopy (SEM)

The cocoa bean shell bioadsorbent was analyzed before and after adsorption of Congo red dye through field-emission scanning electron microscopy on a Jeol JSM-6510LV, Peabody, MA, USA) with a resolution of 1 nm at 20 KeV and 2.5 nm at 5 KeV.

#### 2.4.2. Fourier-Transform Infrared Spectroscopy (FTIR)

The Congo red dye and bioadsorbent were characterized before and after the adsorption process through Fourier-transform infrared spectroscopy (FTIR). For this purpose, a Perkin Elmer Spectrum 100 model was used, which employed a diamond ATR controlled by software for Windows©. The samples of the bioadsorbent were analyzed in the 4000–400 cm^−1^ region, with 16 scans per spectrum and a resolution of 4 cm^−1^. The plots of the FTIR signals and their assignments were made in Origin 8.3.

### 2.5. Experimental Design and Statistical Analysis

According to recent studies, composite central designs have been used in different environmental–technological applications, including heavy metal removal, dye removal, electrocoagulation, and ethanol production [25]. A composite core design with central points and extended with axial points is an experiment designed for the response surface methodology (RSM). The RSM is an experimental and analytical strategy in which a response of interest receives the influence of several independent variables, where the objective is to find the optimal operating conditions of the variables in the system [26]. In the present study, the RSM was used for the optimization of the study variables for the adsorption of Congo red dye on a bioadsorbent prepared from cocoa bean shells suspended in an aqueous medium. Table 1 shows the independent variables, design factors, and their coding. The process intervals were determined according to those used by Cardoso et al. [13]. In this study, a 2^4^ factorial design with eight central points and eight axial points was used.

The axial points were considered; thus, the coded levels of −2 and +2 were defined, to which the points of 40 and 120 mg L^−1^, 0.06 and 0.18 g of adsorbent, 3 and 11 pH, and 4 and 36 h of contact time correspond according to the following expressions:

(2) $$X_{1}=\frac{\mathrm{Dye}\;\mathrm{concentration}-80}{20}$$

(3) $$X_{2}=\frac{\mathrm{Bioadsorbente}-0.12}{0.03}$$

(4) $$X_{3}=\frac{\mathrm{pH}-7}{2}$$

(5) $$X_{4}=\frac{\mathrm{Time}-20}{8}$$

The relationship between the response variables (Adsorption %) and the study factors was expressed in terms of a response polynomial given by

(6) $$\begin{array}{c} Y_{\mathrm{Adsorption}\;\%}=\beta_{0}+\beta_{1}X_{1}+\beta_{2}X_{2}+\beta_{3}X_{3}+\beta_{4}X_{4}+\beta_{12}X_{1}X_{2}+\beta_{13}X_{1}X_{3}+\beta_{14}X_{1}X_{4}+\beta_{23}X_{2}X_{3}+\beta_{24}X_{2}X_{4}+\\ \beta_{34}X_{1}X_{2}+\beta_{11}X_{1}^{2}+\beta_{22}X_{2}^{2}+\beta_{33}X_{3}^{2}+\beta_{44}X_{4}^{2}+\varepsilon , \end{array}$$

where Y_Adsorption %_ is the response; X_1_, X_2_, X_3_, and X_4_ are lineal factors; X_12_, X_13_, X_14_, X_23_, X_24_, and X_34_ are interaction factors; and $X_{1}^{2},{\;X}_{2}^{2},{\;X}_{3}^{2},{\;\mathrm{and}\;X}_{4}^{2}$ are quadratic factors. β_0_, β_i_ (i = 1, …, 4), and β_ij_ (i = 1, …, 4; j = 1, …, 4) are the regression coefficients of the polynomial. An analysis of variance (ANOVA) and multiple linear regression were applied and performed in MATLAB R2017b.

## 3. Results

### 3.1. Characterization of the Cocoa Bean Shell Bioadsorbent

#### 3.1.1. Scanning Electron Microscopy (SEM)

**Figure 1:**
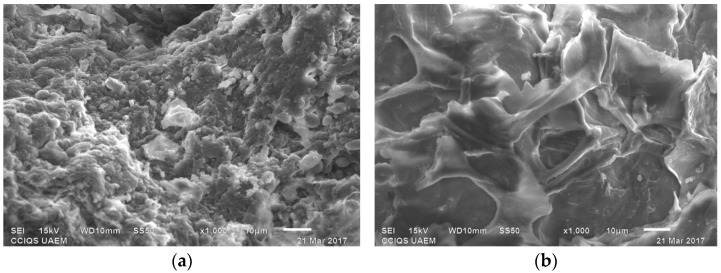
SEM photograph of cocoa bean shell powder at 1000× before (**a**) and after (**b**) the adsorption process.

Figure 1 shows the images obtained by applying scanning electron microscopy to the bioadsorbent before (Figure 1a) and after (Figure 1b) the adsorption of the dye at a magnification of ×1000.

It was observed that the surface of the bioadsorbent before the adsorption process showed a highly rough and heterogeneous surface with a high porosity and spheroidal particles of micrometric size. However, after the process of adsorption of the dye, it was found that the morphology of the material underwent a total transformation, presenting a homogeneous surface with little porosity. This was a consequence of the superficial adsorption of the Congo red and the changes caused by the contact with the water of the solution, which generated a swelling of the fibers, resulting in the appearance of a completely smooth texture.

#### 3.1.2. Fourier-Transform Infrared Spectroscopy (FTIR)

**Figure 2:**
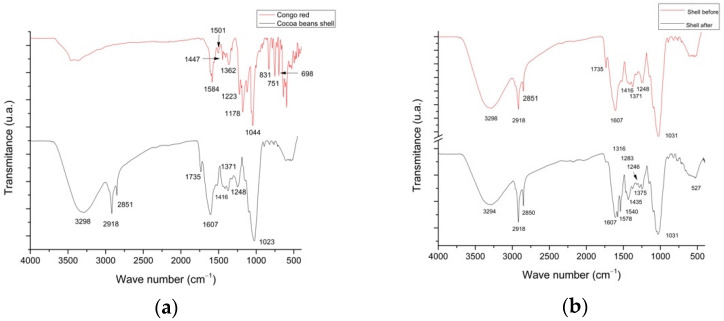
FTIR spectra of Congo red dye and cocoa bean shells (**a**) and of cocoa bean shells before and after the Congo red dye adsorption process (**b**).

The FTIR technique was used to examine the prevailing functional groups in the bioadsorbent before and after adsorption of Congo red dye. Figure 2 shows the vibrational FTIR spectra of the Congo red dye and the cocoa bean shell powder both before and after the adsorption of the dye. The FTIR spectrum of the Congo red dye (Figure 2a) showed a broad signal without a defined peak between 3463 and 3372 cm^−1^; according to Lafi et al. [27], this would be associated with OH and NH_2_ groups. The peaks identified at 1584, 1447, and 1362 cm^−1^ were similar to those reported by Lafi et al. [27] and correspond to amino groups, -N-H bending, and -S=O stretching vibrations, respectively.

In the cocoa bean shells, the intense absorption band at 3298 cm^−1^ was assigned to the stretching of the OH group [13], while the signals at 2918 and 2850 cm^−1^ corresponded to the stretching of the CH groups, which is characteristic of CH_2_ methylene groups. These two groups prevailed in the infrared spectrum of the bioadsorbent sample without alterations in their intensities after Congo red dye adsorption (Figure 2b). This showed that these groups did not participate in the adsorption process. The bands at 1735 cm^−1^ corresponded to the C=O stretching of the carbonyl group of carboxylic acids, while the band at 1607 cm^−1^ corresponded to the vibrational modes of the aromatic ring [28], where amides, aldehydes, ketones, carboxylic acids, and flavonoids were located. According to Cardoso et al. [13] and Lozada et al. [29], some of these bands may overlap due to the bending vibrations of the C–H bonds of alkyl groups. Two absorption bands, one at 1248 cm^−1^ and a very intense one at 1031 cm^−1^, were assigned to the C–O stretching of phenolic compounds, corresponding to cellulose, lignin, and alcohols [30,31]. The signals between 607 and 527 cm^−1^ corresponded to bending outside the plane of the C–H bond [28], confirming the presence of an aromatic ring with respect to the 1607–1416 cm^−1^ assignment. In the study that Cardoso et al. [13] conducted with Cupuaçu shells (Theobroma grandiflorum), they reported that the interaction of Congo red with the adsorbent matrix was evidenced by signals corresponding to the OH bonds of phenolic compounds and alcohols that were present in the lignin structure. This behavior was also observed in this study, as shown in Figure 2, in terms of the size, intensity, and length of the bands before and after (1416–1023 cm^−1^ and 1031 cm^−1^), thus confirming the anchorage of the dye in the adsorbent. On the other hand, in the present study, changes were observed before and after the adsorption (Figure 2b) process, with particular evidence in the signals at 1607, 1578, and 1540 cm^−1^, which are intervals that are assigned to aromatic ring vibration modes, indicating the participation of aldehyde groups found in the scale and primary amines; these are the key points in the anchorage between the adsorbent and Congo red dye.

### 3.2. Adsorption of Congo Red Dye by the Bioadsorbent from Cocoa Bean Shells

The results of the study on the adsorption of Congo red by the bioadsorbent obtained from cocoa bean shell are shown in Table 2.

The adsorption values were found to be above 70% for all of the runs evaluated in the design. Run 11 showed that the minimum adsorption value of the dye was 74.29%, while the maximum was found in Run 24 with 89.86%. The results show that, in spite of the changes in the experimental levels of the different factors, an adsorption percentage higher than 90% was not reported.

The results of the statistical analysis are shown in Table 3 and Table 4. The proposed statistical model was significant for the variable corresponding to the percentage of Congo red adsorption; therefore, it can be used for the purpose of evaluating the effects and optimization.

The analysis of variance (ANOVA) is summarized in Table 4. Linear and quadratic effects can be identified as the most significant values for dye adsorption. A value of R^2^ = 0.84 was obtained, which implies that the model explained 84% of the variability in the adsorption percentage, and R^2^_adjusted_ = 0.709, which is the modified R^2^ for the terms of the model. These coefficients of determination (R^2^ and R^2^_adjusted_) are both required to have values greater than 0.7 or 70% according to Gutiérrez and de la Vara [32]. Table 3 presents the regression coefficients together with the standard errors, statistical *t* tests, and probability values. The significant factors were X_1_, X_2_, X_3_, X_4_, and X_2_^2^ (X_1_ = dye concentration, X_2_ = amount of adsorbent, X_3_ = pH, and X_4_ = contact time), which presented a probability level of 5% (*p* < 0.05); thus, these factors had an effect on the adsorption of the Congo red dye. The interaction coefficients (X_1_X_2_, X_1_X_3_, X_1_X_4_, X_2_X_3_, X_2_X_4_, and X_3_X_4_) and the quadratic coefficients (X_1_^2^, X_3_^2^, and X_4_^2^) did not have any significant effects (*p* > 0.05).

The response surface methodology was used with the 2^4^ factorial design with central points and axial points. This is an optimization technique for obtaining the maximum desired response [33]. The MATLAB R2017b software was used to perform an analysis of variance (ANOVA) and a multiple linear regression analysis, in addition to creating response surface plots. The number of runs generated from the design is a function of the degrees of freedom needed to solve a second-order polynomial and to be able to express the relationship between the independent variables (factors) and the dependent variable for the case under study (% adsorption of Congo red dye). Equation (7) shows the development of the polynomial considering the linear, interaction, and second-order effects:

(7) $$\begin{array}{c} Y_{\%\mathrm{Adsorption}}=83.70-2.14X_{1}+1.20X_{2}-1.06X_{3}+1.33X_{4}+0.62X_{1}X_{2}+0.20X_{1}X_{3}-0.75X_{1}X_{4}+0.88X_{2}X_{3}-\\ 0.64X_{2}X_{4}-0.04X_{3}X_{4}-0.42X_{1}^{2}-1.19X_{2}^{2}+0.56X_{3}^{2}+0.21X_{4}^{2}. \end{array}$$

**Figure 3:**
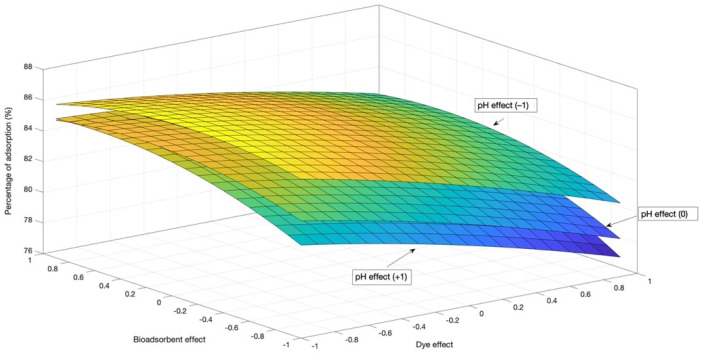
Estimated response area for the percentage of Congo red dye adsorption by the cocoa bean shell powder.

Equation (7) makes it possible to reproduce the behavior of the response variable (adsorption percentage) with respect to the four study factors. This expression was used to generate Figure 3, which shows the response surface curve.

In Figure 3, the factor of contact time remains constant. The quadratic effect of bioadsorbent × bioadsorbent results in the estimation of the response surface by displaying the curvature.

### 3.3. Effect of pH

The statistical analysis applied to the adsorption of the dye by the bioadsorbent showed a negative linear effect of solution pH on the retention capacity of the bioadsorbent. Mane and Babu [18] and Zhang et al. [34] reported the pH dependence of dye adsorption; an 80% adsorption of Congo red dye was obtained in a pH range of 4–10, so this pH range was used in our study, and the same behavior was found in the different runs evaluated. The experimental results showed that adsorption decreased with increases in pH (*p* < 0.05) due to the effect of the combination of the simultaneously evaluated factors. The decrease in the capacity for the adsorption of Congo red dye when the pH of the solution increased above four was attributed to the fact that this parameter affected the degree of ionization and the specificity of the adsorbent, changed its surface charge, and made the adsorption dependent on the electrostatic attraction between the Congo red dye molecules and the bioadsorbent material [20,35,36].

At an acidic pH, the concentration of H+ ions in the solution increased, and they interacted with the surface of the bioadsorbent, acquiring a positive charge. However, at an alkaline pH, there was competition between OH- ions and the anionic species of the dye for available adsorption sites [7,20,36]. The increase in the dye adsorption capacity in the pH range of 2.0–4.0 was observed and attributed to the formation of a Schiff base between a dialdehyde group and the primary amine groups. This phenomenon explains the observations in this study, as the cocoa bean shell is a material that possesses large amounts of compounds that have aromatic rings and interact with Congo red dye, as was verified in the FTIR spectra before and after the adsorption process. Similarly, other reports with lignocellulosic materials have presented similar results in terms of the effect of pH, such as in the case of jujuba seeds, cashew nut shells, sunflower seed hulls, soy meal hulls, bagasse fly ash, jute stick powder, and other adsorbent materials, such as activated carbon, Ca-bentonite, perlite, rice shell ash, kaolin, and zeolite [20,34].

### 3.4. Effect of the Bioadsorbent Concentration

In previous studies of dye adsorption by Mane and Babu [18] and Zhang et al. [34], it was observed that an increase in the mass of the adsorbent used causes an increase in the adsorption capacity of the adsorbent until an inflection point where equilibrium is reached, after which the adsorption of the molecule under study may even be decreased. This behavior has made it necessary to evaluate the retention capacity of each adsorbent in order to find the adsorption equilibrium for each material. The surface area plays a preponderant role in the adsorption phenomenon because a high specific area represents a greater number of available sites for adsorption. Therefore, this property of the adsorbent material plays a fundamental role in its capacity to retain contaminant molecules [18,34,36,37].

In this work, the specific area of the bioadsorbent was not measured, but the effect of the adsorbent concentration (mass of 0.06 to 0.18 g) was studied. The changes in dye removal were governed by the effects of the mass of adsorbent that was used in the present study; this factor was highly statistically significant (*p* < 0.01) with respect to the concentration of Congo red dye adsorbed, with a positive linear effect. That is, this effect is shown as an increase in dye adsorption as a function of the amount of adsorbent. However, no saturation was observed because the maximum adsorption occurred at the highest adsorbent level, indicating that the bioadsorbent had a large number of adsorption sites available and that they were not saturated with dye molecules. The increase and decrease in the adsorption capacity of Congo red dye as a function of adsorbent mass were also reported in studies performed with spent mushroom, eucalyptus wood sawdust, and sugarcane bagasse [18,34,36,37].

### 3.5. Effects of the Initial Dye Concentration and the Contact Time

The contact time showed a positive linear effect in this study, which indicated that adsorption increased as a function of time (12–28 h). This phenomenon indicates that equilibrium was not reached under the conditions in which the study was conducted due to the high adsorption capacity of the bioadsorbent. Several studies have shown that 4 h of contact is sufficient for reaching the adsorption equilibrium; however, this depends on the transfer mechanisms involved in the adsorption process, as demonstrated by Cardoso et al. [18], who used cupuassu to show that there is more than one kinetic stage (adsorption rate) depending on the type of dye used.

The effect of the initial concentration of Congo red (40–120 mg L^−1^) in the adsorption experiments using cocoa bean shells as a bioadsorbent presented a negative linear effect. This negative effect implies that, as the initial concentration of the dye increases, the adsorption capacity of the bioadsorbent decreases; this behavior was also described in a study of RC adsorption, which concluded that the dye adsorption phenomena are directly dependent on the concentration of the solute [18,36]. The above is because the adsorption capacity at equilibrium increases as the initial concentration of the dye increases, with the mechanism of resistance to Congo red removal being the controller of the process.

### 3.6. Optimization of the Adsorption Process

By means of the MSR, the effects of the factors were evaluated and a polynomial equation was generated in order to reproduce the percentage of Congo red dye degradation; from this, the best selection of the variables according to a defined criterion was found in such a way that was independent of the multiplicity of solutions. For the present study, the focus was on maximizing the percentage of Congo red dye adsorption by the bioadsorbent to 100% without restrictions. Based on the method of desirability, a theoretical optimum combination of the four study factors was found (Table 5).

From the optimal conditions, which are proposed in their coded form, the natural conditions were established from Equations (2)–(5) for validation. The natural conditions in this study were 40 mg L^−1^ (dye), 0.1168 g (bioadsorbent), 3 (pH), and 36 h (time). From the experimental results obtained by applying the optimum conditions, the model was validated, obtaining 95.79% of dye adsorption on the bioadsorbent prepared from cocoa bean shells.

### 3.7. Comparison with Other Dye Adsorption Studies

The wide diversity of agricultural solid waste and its use for the removal of colorants in effluents has allowed it to be classified into four categories [38]: powders, skins, fibers, and shells. The different methods for the preparation and use of waste as adsorbents make the comparison between materials and studies difficult. Materials are usually reported to have good adsorption properties, and their properties can be improved as a function of chemical and physical treatments, such as conversion into activated carbon, chemical treatments for functionalization, or direct ultrasound on the raw materials. With the aim of comparing our study with others, four CR adsorption studies that used wood sawdust, cane bagasse, defatted soy residue, and apple seeds were considered [7,18,34,36]. These studies have a common point in that they considered kinetic and thermodynamic studies, unlike our study, which had a statistical design, so the conclusions of the studies differed. All of the studies considered the initial concentration of the dye and the initial pH as study factors, but they differed in that some studies also considered the effects of the temperature, adsorbent mass, contact time, and surface area of the adsorbent. On the other hand, wood sawdust was treated with NaOH, while our study and the others did not perform chemical treatments. However, the initial concentrations differed, as well as the surface contact areas and adsorbent masses. In particular, the effects of the study factors were greater; in terms of the time taken to reach the adsorption equilibrium, the studies coincided with a time of 4 h using wood sawdust and defatted soy, while with apple seed, equilibrium was reached at 90 min. However, in our study, the interest was in maximizing the adsorption based on a design that considered the study factors simultaneously and not independently, as reported by the studies cited by the other authors. Therefore, there was a lack of a common platform on which to group the different classical treatment methods with different approaches. Therefore, the materials are promising, and for their use on different scales, it is necessary to evaluate their performance and cost for industrial applications.

## 4. Conclusions

The purpose of this study was to prove the potential of an available agricultural waste, cocoa bean shells, as an adsorbent. A response surface design allowed the evaluation of the effects of four factors on the percentage of Congo red dye adsorption using cocoa bean shell powder. The second-order statistical model was significant (*p* < 0.05), and the goodness-of-fit test showed that a non-linear model was adequate for reproducing the response variable. The factor with the greatest influence was pH, which presented a positive linear effect and a negative non-linear effect. This study made it possible to find the optimal operating conditions for the variables of dye concentration (40 mg L^−1^), amount of bioadsorbent (0.1168 g), pH value (3) of the solution, and processing time (36 h); under these conditions, a percentage of adsorption of Congo red dye of 95.79% was achieved. By means of infrared spectroscopy, it was determined that the dye was anchored on the surface of the bioadsorbent—in particular, it was linked to the OH groups of the phenolic compound molecules and alcohols—and aldehyde groups and primary amines were also involved. With this evidence, it was confirmed that the cocoa bean shell bioadsorbent represents an alternative for the removal of contaminants in water. Future studies will involve the evaluation of the recovery of the dye, as well as tests in continuous systems in which the shells are packed and there is a continuous flow of dye in an aqueous medium. Finally, tests will be developed on a pilot scale to evaluate the statistically optimal design and the savings of time, costs, effort, and labor in comparison with the conventional method.

## Figures and Tables

**Table 1:** Intervals of experimental conditions of the variables and levels for the process of adsorption of Congo red dye.

Adimensional Factor	Name	Low Level	Central Level	High Level	Coded Low	Coded Central	Coded High
X_1_	Dye [mg L^−1^]	60	80	100	−1	0	+1
X_2_	Bioadsorbent (g)	0.09	0.12	0.15	−1	0	+1
X_3_	pH	5	7	9	−1	0	+1
X_4_	Time (h)	12	20	28	−1	0	+1

**Table 2:** Runs for the experiment on the process of adsorption of Congo red dye.

Run	Coded Variables				Natural Variables				% Adsorption
	X_1_	X_2_	X_3_	X_4_	Dye (mg L^−1^)	Bioadsorbent (g)	pH	Time (h)	
1	−1	−1	−1	−1	60	0.09	5	12	85.32
2	−1	−1	−1	1	60	0.09	5	28	87.82
3	−1	−1	1	−1	60	0.09	9	12	78.48
4	−1	−1	1	1	60	0.09	9	28	85.43
5	−1	1	−1	−1	60	0.15	5	12	85.22
6	−1	1	−1	1	60	0.15	5	28	87.92
7	−1	1	1	−1	60	0.15	9	12	84.57
8	−1	1	1	1	60	0.15	9	28	85.32
9	1	−1	−1	−1	100	0.09	5	12	80.47
10	1	−1	−1	1	100	0.09	5	28	79.96
11	1	−1	1	−1	100	0.09	9	12	74.29
12	1	−1	1	1	100	0.09	9	28	77.41
13	1	1	−1	−1	100	0.15	5	12	80.90
14	1	1	−1	1	100	0.15	5	28	83.42
15	1	1	1	−1	100	0.15	9	12	84.01
16	1	1	1	1	100	0.15	9	28	79.74
17	−2	0	0	0	40	0.12	7	20	85.56
18	2	0	0	0	120	0.12	7	20	79.79
19	0	−2	0	0	80	0.06	7	20	77.84
20	0	2	0	0	80	0.18	7	20	81.34
21	0	0	−2	0	80	0.12	3	20	87.58
22	0	0	2	0	80	0.12	11	20	85.74
23	0	0	0	−2	80	0.12	7	4	80.67
24	0	0	0	2	80	0.12	7	36	89.86
25	0	0	0	0	80	0.12	7	20	84.76
26	0	0	0	0	80	0.12	7	20	81.07
27	0	0	0	0	80	0.12	7	20	83.43
28	0	0	0	0	80	0.12	7	20	84.72
29	0	0	0	0	80	0.12	7	20	83.50
30	0	0	0	0	80	0.12	7	20	84.33

**Table 3:** Multiple regression analysis of the adsorption percentage of Congo red dye in the cocoa bean shell powder.

Parameters	Estimated Coefficient	Standard Error	*t* Test Statistics	*p*-Value
Constant	83.704	0.6608	126.67	9.77 × 10^−27^
X_1_	−2.1421	0.3815	−5.6147	3.09 × 10^−5^ *
X_2_	1.2054	0.3815	3.1594	0.0057 *
X_3_	−1.0612	0.3815	−2.7816	0.0127 *
X_4_	1.3388	0.3815	3.5091	0.0026 *
X_1_X_2_	0.6226	0.4672	1.3325	0.2002
X_1_X_3_	0.2001	0.4672	0.4283	0.6737
X_1_X_4_	−0.7536	0.4672	−1.6129	0.1251
X_2_X_3_	0.8845	0.4672	1.893	0.0755
X_2_X_4_	−0.6461	0.4672	−1.3829	0.1846
X_3_X_4_	−0.0404	0.4672	−0.0865	0.9320
X_1_^2^	−0.4283	0.3439	−1.2456	0.2298
X_2_^2^	−1.1998	0.3439	−3.4888	0.0028 *
X_3_^2^	0.5667	0.3439	1.648	0.1177
X_4_^2^	0.2179	0.3439	0.6337	0.5346

* Significant values (*p* < 0.05).

**Table 4:** Analysis of variance (ANOVA) of the adsorption percentage of Congo red dye in the cocoa bean shell powder.

	Sum of Squares	Degrees of Freedom	Average Squares	F	*p*-Value
Total	371.91	31	11.997		
Model	312.52	14	22.323	6.3899	0.0002
Linear	215.05	4	53.762	15.389	1.71 × 10^5^ *
Non-linear	97.471	10	9.7471	2.7901	0.0302 *
Residue	59.389	17	3.4935		
Lack of adjustment	49.338	10	4.9338	3.4361	0.0574
Pure error	10.051	7	1.4359		

* Significant values (*p* < 0.05).

**Table 5:** Optimal operating conditions for the adsorption of Congo red dye.

Factor	Low	High	Optimal
Dye concentration	−2.0	2.0	−2.0
Bioadsorbent	−2.0	2.0	0.79
pH	−2.0	2.0	−2.0
Time	−2.0	2.0	2.0

## Data Availability

The data presented in this study are available on request from the corresponding author.

## Abbreviations

ANOVA	Analysis of variance
Bioadsorbent	Mass of the bioadsorbent (g)
c_i_	Initial concentration (mg L^−1^)
c_f_	Final concentration (mg L^−1^)
CH_2_	Methylene functional group
CH	Carbon–hydrogen bond
C=O	Carbonyl functional group
dm	Dry matter
Dye	Concentration (mg L^−1^)
FT IR	Fourier-transform infrared spectroscopy
NH_2_	Amino functional group
OH	Hydroxyl functional group
pH	pH value (3–11)
RSM	Response surface methodology
R^2^	Coefficient of determination
R^2^(adjusted)	Adjusted coefficient of determination
SEM	Scanning electron microscopy
S=O	Sulfate functional group
Time	Time (h)
X_1_	Adimensional linear factor for dye
X_2_	Adimensional linear factor for bioadsorbent
X_3_	Adimensional linear factor for pH
X_4_	Adimensional linear factor for time
% *A*	Adsorption percentage of Congo red dye (%)
*Y_Adsorption %_*	Response variable of adsorption percentage (%)
Greek symbols	
β	Regression coefficient
ε	Random error
Subindex	
i,j	Index notation of the factors or coefficient
